# Community influences on modern contraceptive use among young women in low and middle-income countries: a cross-sectional multi-country analysis

**DOI:** 10.1186/s12889-018-5331-y

**Published:** 2018-04-02

**Authors:** Massy Mutumba, Eliud Wekesa, Rob Stephenson

**Affiliations:** 1 Department of Health Behavior and Biological Sciences, University of Michigan School of Nursing, 3177, 400 N.Ingalls Bldg, Ann Arbor, MI 48109-5482 USA; 2 Department of Humanities and Social Sciences, South Eastern Kenya University, Kitui, Kenya

## Abstract

**Background:**

Despite investment in family planning programs and education, unmet need for family planning remains high among young women (aged 15–24) in low and middle-income countries, increasing the risk for unwanted pregnancies and adverse social and reproductive health outcomes. There is a dearth of cross-national research that identifies the differential impact of community level factors among youth in low and middle-income countries (LMICs), which is imperative for the design of structural level interventions aimed at increasing family planning use.

**Methods:**

Grounded in the socio-ecological framework, this paper utilizes Demographic and Health Survey (DHS) from 52 LMICs to examine the influence of community level reproductive, gender, fertility, literacy and economic indicators on modern contraceptive use among female youth. Analyses are conducted using multi-level logistic regressions with random community-level effects.

**Results:**

Our findings highlight the positive influence of community level education attainment and negative influence of gender and fertility related norms on young women’s contraceptive use. Additionally, increased exposure to mass media did not positively influence young women’s uptake of modern contraceptive methods.

**Conclusions:**

Taken together, findings indicate that young women’s contraceptive decision-making is greatly shaped by their social contexts. The commonalities and regional variations in community level influences provide support for both structural level interventions and tailored regional approaches to family planning interventions.

## Background

Family planning is one of the most important health interventions of the twentieth century [1]. It enables women to plan their births and determine the number of children to have. Its use has far-reaching benefits for individuals, couples, households, communities, and society at large, including: maternal and child health improvements, educational advances, reduction of poverty and empowerment of women. Yet despite these benefits and ongoing efforts to expand its access, contraceptive use is still low and unmet need for family planning is high in developing countries. Currently, an estimated 225 million women in the developing world have unmet need for family planning i.e. they wish to avoid or delay pregnancy but are not using any contraceptive method. This exposes them to unintended (mistimed and unwanted) pregnancies, maternal and childhood deaths, morbidity and unsafe abortions [2]. It is estimated that eliminating unmet need for FP in developing countries could avert up to 30% of pregnancy related deaths [3].

Contraceptive use among young women (aged 15–24), whether married or unmarried, is lower than among relatively older women in the developing world. Consequently, over 39.5% of young unmarried women and 25.9% of young married women in low and middle-income countries have an unmet for family [4]. Unmet need is highest among young women in Africa, primarily, the West and Central African regions (29.3% among young married women and 41.7% among young unmarried women), in the Middle East and North Africa (10.8%). Among individual countries, it is highest in young married women in Senegal (69.5%) and lowest in Ukraine (7.3%). For the unmarried young women, it is highest in Ghana (45.7%) and Haiti (44.8%) and lowest in Egypt (8.8%) and Indonesia (8.0%) [5]. The high unmet need levels underscore the importance of understanding the factors that promote or hinder utilization of contraceptives among young women.

There are several gaps in the literature on contraceptive use among young women in the developing world. First, the majority of studies that inform our understanding of contraceptive use among young women have focused on individual and institutional level factors at the expense of community factors. As such the extent to which community characteristics affect contraceptive use among women is not well understood. There is growing evidence that young people are strongly influenced by their environment [3], thus suggesting that community characteristics may influence young women’s reproductive behavior [6, 7]. An emerging body of evidence has reported associations between community characteristics (e.g. educational attainment of women, community norms related to marriage and childbearing, women participation in household decision-making, attitudes towards intimate partner violence) and contraceptive use [8–10] and unmet need for family planning [11].

The growth of interest in community level factors on FP use notwithstanding, the majority of studies largely combine younger and older women. However, there is reason to believe that these community level factors may operate differently in younger and older women. For example, a prior study conducted among Ugandan women reported on the age differences in the influence of individual level factors such as exposure to mass media and perceptions of distance to the clinic on contraceptive use [12]. In Zimbabwe, Ngome and Odimegwu found that adolescents aged 15–19 years residing in communities with a higher mean number of children ever born or a higher mean number of years of education were less likely to use contraceptives [7].

Additionally, the majority of existing studies on community level influences on contraceptive use have been focused on women in Africa. The extent to which these findings could be generalized to women in other low and middle-income countries is less clear. The objective of this paper is twofold: to examine the global commonalities in community level influences on young women’s contraceptive use, and (2) assess for regional variations in the influences of these community level factors on young women’s contraceptive use. This information will inform global and regional strategies for promoting modern contraceptive uptake among young women.

### Conceptual framework

Bronfenbrenner’s socio-ecological theory provides a relevant framework for understanding how community level factors may shape young women’s contraceptive behavior [13, 14]. This conceptual framework proposes that a woman’s contraceptive use behavior is shaped by her individual-level attributes (e.g. age, education attainment, household wealth, place of residence) and household level factors [12, 15–18] and the community context in which she lives [8–10, 19, 20]. Community characteristics may influence contraceptive use through a multitude of pathways. Community demographics (age at first marriage and sexual debut) may be closely aligned with fertility norms or expectations of ideal family size [8–10]. Mass media exposure may increase social interaction, which facilitates diffusion of knowledge on FP, dispelling myths and misconceptions around FP and overcoming cultural barriers to FP use [18, 21, 22]. Education is associated with increased age at first marriage and age at first birth, both of which have been associated with contraceptive use [23]. Social development indicators such as household wealth and education attainment in the community facilitate FP access, enhance autonomy in seeking FP and change perceptions of the opportunities and costs associated with childbearing, thus reducing the desire for larger family sizes [22].

Gender norms and inequities including gendered imbalances in women’s access to education impact women’s decision-making autonomy and exposure to intimate partner violence, thereby influencing their access and utilization of family planning services [11, 17]. These community level factors may operate differently among young people because youth have unique social and developmental needs e.g. social roles and status, perceived risks, access to information, and decision-making autonomy that may shape their uptake of contraceptives in ways that differ meaningfully from older women [24–26]. For example, young women are subject to the authority of adults and institutions such as schools, religious and cultural organizations, and health care institutions that are vested in shaping and regulating their behavior [17, 18, 27].

## Methods

### Data

The data utilized in these analyses are drawn from Demographic and Health Surveys (DHS) conducted between 2008 and 2016 in 52 countries, in the African region (32), South East Asia region (6), Eastern Mediterranean region (5), Western Pacific region (2), European region (5) and the Americas (8). The DHS collect data from reproductive age women (ages 15–49). Sampling is based on a standardized two-stage sampling design. Within each country, primary sample units (PSUs) are selected using the most recent census in each country as the sample frame. Then, approximately 20 to 30 households are selected from a listing of households in each PSU. This standardization allows for comparability for indicators across countries. These analyses focus on young women aged 15–24 years, irrespective of their marital status (*N* = 305, 471). Table 1 provides a descriptive summary of all the countries included in the analyses.

**Table 1:** Sample size and descriptive statistics of young women across regions and country

Country	Sample size (N)	Modern contraceptive prevalence rate	Country	Sample size (N)	Modern contraceptive prevalence rate
Burkina Faso	6592	12.0	Uganda	3692	12.4
Benin	5742	9.4	Zambia	6726	20.5
Burundi	4231	6.0	Zimbabwe	3938	29.1
Democratic Republic of Congo	7661	6.5	Afghanistan	7912	10.5
Congo	3963	22.4	Albania	2471	3.4
Cote d’Ivoire	3984	13.7	Armenia	1898	4.3
Cameroon	6708	16.8	Bolivia	6335	13.9
Ethiopia	6857	12.1	Cambodia	6044	12.0
Gabon	3407	24.2	Columbia	12,400	43.0
Ghana	3327	13.6	Dominican Republic	3545	31.7
Guinea	3608	7.6	Egypt	3789	34.6
Kenya	11,483	20.5	Guatemala	10,597	15.8
Comoros	2282	6.1	Guyana	1791	21.8
Liberia	3499	19.7	Haiti	6272	14.4
Lesotho	2842	35.5	Honduras	9347	24.5
Madagascar	6935	14.4	Indonesia	13,796	18.5
Mali	3798	8.8	Jordan	1429	26.0
Malawi	10,367	30.4	Kyrgyz	3105	7.0
Mozambique	5533	14.4	Maldives	1510	15.4
Nigeria	14,619	9.3	Myanmar	3728	15.4
Niger	3869	8.6	Nepal	5071	10.8
Namibia	3577	39.7	Pakistan	2615	13.7
Rwanda	5252	9.9	Peru	8078	22.3
Sierra Leone	6739	24.8	Philippines	6070	8.8
Senegal	3756	6.8	Tajikistan	3901	3.5
Sao Tome	987	23.8	Timor-Leste	5566	3.1
Swaziland	2292	27.6	Ukraine	1807	26.2
Togo	3337	15.1	Yemen	11,560	19.1
Tanzania	5399	15.8	Bangladesh	5184	51.1

### Measures

The primary outcome measure in these analyses was use of a modern contraceptive method (i.e. pills, injectables, implants, male or female condoms, male or female sterilization, foam or jelly). The DHS does not collect data on community level variables. Similar to previous studies that have examined community level influences on reproductive level [8, 10, 19, 20], community level variables were created by individual level data of all community members in a primary sampling unit (PSU). PSUs in rural areas may be a closer approximation of the actual community compared to urban areas where the dense clustering of communities could result in artificial demarcations of communities within PSUs. To account for potential differences in the construction of PSUs between urban and rural areas, our analyses include an indicator for urban or rural place of residence). The community level variables were estimated on all reproductive age women (ages 15–49) in order to provide a measure of the average behavior and beliefs of all women in the community, and not just the young women.

This analysis is necessarily limited to including the following community level indicators that can be created from individual data in the DHS: age (at sexual debut, marriage and first birth); desired fertility; mass media exposure; decision making autonomy; gender-based violence, education attainment and household wealth in the community. Multilevel modeling technique helps in analyzing hierarchically clustered data to estimate variation between communities. A detailed description of the operation definitions of the community level variables is provided in Table 2. The choice of community level characteristics was based on findings from prior studies on community level influences [8, 10, 19], and these included: *community demographics* (i.e. mean education attainment of women, mean exposure to mass media, and mean HIV knowledge score, mean household wealth index score); *fertility norms* (i.e. mean ages at marriage, first birth and sexual debut; and mean ideal family size); and *gender norms* (e.g. mean household decision-making autonomy and mean attitudes towards intimate partner violence).

**Table 2:** Operational description of community level variables included in the analyses

Community demographic factors	
Mean education attainment	Computed as the mean number of education years among women aged 15–49
Community household wealth index factor	Computed from the household wealth index factor score as mean community.
Mean HIV prevention knowledge	Computed as a mean of a 7-item scale assessing HIV knowledge. Scale items include: one item on HIV awareness (ever heard of HIV/AIDS), three items about HIV risk reduction strategies (abstinence, consistent condom use, and having one with no other partners), and three items on HIV transmission (getting AIDS from mosquitoes, getting AIDS from sharing food with an infected person, and whether a healthy-looking person can have AIDS). Responses are measured on a 3-point scale (yes/ no/ don’t know). In these analyses, the option “don’t know” was recoded into “No”). A higher score indicates higher HIV knowledge.
Community exposure to mass media score	Computed as the mean score on three items assessing exposure to mass media: frequency of listening to radio, watching TV and reading newspapers. Responses are measured on a 3-point scale; a higher score indicates greater exposure to mass media.
Community fertility norms	
Mean age at marriage	Mean age at marriage among all women aged 15–49
Mean age at first birth	Mean age at first birth among all women aged 15–49
Mean age at sexual debut	Mean age at first sex among all women aged 15–49
Mean ideal family size	Mean ideal number of children among all women aged 15–49
Community gender norms and inequity	
Mean household decision making autonomy	Mean score on the household decision-making autonomy index in the community. Household decision-making was computed from a five-item scale assessing final say on respondents’ own health care, making large purchases, household purchases for daily needs, visits to family or relatives, and food cooked each day. A higher score indicates higher decision-making autonomy.
Mean attitudes towards intimate partner violence	Mean score on the violence justification index scale in the community. Violence justification was computed from a five-item scale with responses measured on a 5-point scale; a higher score indicates higher decision-making autonomy. Items include: going out without telling partner, neglecting the children, arguing with partner, refusing to have sex with the partner, and burning food.

### Data analysis

Given the nested data structure (i.e. women nested in communities), multi-level analytic approach was utilized to examine the influence of the community level factors on contraceptive use among young women, after controlling for women’s individual and household level characteristics. The analyses were conducted in STATA v14.0. software package. The analyses were unweighted because the DHS do not include higher level weights (e.g. PSU) that are required for multi-level analyses. However, this methodology allows for specifically of nested random effects.

To examine the global commonalities in community level influences on young women’s contraceptive use, all the data were pooled into a global dataset. Unique country level PSUs was created by combining the country, region and PSU identifiers. A multi-level logistic regression model with random community level (i.e. PSU) effects and a fixed effect country variable was used to examine global community level influences on young women’s contraceptive use. This model controlled for young women’s individual socio-demographic characteristics i.e. age, education level, current marital status, place of residence, household wealth, exposure to mass media and living number of children. To assess for regional variations in the influences of these community level factors on young women’s, a series of multi-level logistic regression models with random community level effects and fixed effect country level factor were fit for each World Health Organization (WHO) region. These models also controlled for young women’s individual socio-demographic characteristics.

## Results

### Descriptive statistics

As shown in Table 1, the prevalence of modern contraceptive use (mCPR) among young women ranged from 3.1% in Timor-Leste to 51.1% in Bangladesh. The overall mCPR for the global sample was 17.8%.

### Individual level influences on contraceptive use

Our findings on the associations between individual level factors and modern contraceptive use (not presented) were consistent with prior research. Briefly, young women who were older, those with higher education, those living in wealthier households, living in urban areas, greater exposure to mass media and a higher number of living children were more likely to be using a modern contraceptive method. A few variations emerged in the region specific models. In the Americas and Western Pacific regions, young women living in rural areas were more likely to be using a modern contraceptive method. Increased exposure to mass media was associated with lower odds for modern contraceptive use among young women in the African region and Western Pacific region.

### Community-level influences on contraceptive use

#### Global patterns

Table 3 presents the results of the global model on community level influences on modern contraceptive use among young women. The findings indicate that young women living in communities with a higher age at first marriage [AOR: 1.08], greater autonomy in household decision-making [AOR: 1.02], higher education attainment of women in the community [AOR: 1.05] and those living in wealthier communities [AOR: 1.00] were more likely to be using a modern contraceptive method. Conversely, young women living in communities with a lower mean age at first birth [AOR: 0.91], lower mean age at sexual debut [AOR: 0.79], higher ideal number of children [AOR: 0.65], greater exposure to mass media [AOR: 0.96] and more egalitarian attitudes towards intimate partner violence [AOR: 0.95] were less likely to report using a modern contraceptive method.

**Table 3:** Results of multi-level analyses examining the effect of community level factors on modern contraceptive use among young women in low- and middle income countries

Community level factor	Adjusted Odd ratio (AOR) and *p*-value	95% Confidence interval (CI)
Community mean age at marriage	1.078***	[1.07,1.09]
Community mean age at first birth	0.912***	[0.90,0.93]
Community mean age at sexual debut	0.799***	[0.79,0.81]
Community mean ideal number of children	0.645***	[0.63,0.65]
Community mean mass media exposure score	0.956***	[0.95,0.97]
Community mean household decision-making autonomy score	1.020***	[1.01,1.03]
Community mean attitudes towards intimate partner violence score	0.946***	[0.94,0.95]
Community mean years of completed education	1.045***	[1.04,1.05]
Community mean household wealth score	1.000	[1.00,1.00]

Analyses adjusted for age, education level, current marital status, type of place of residence, household wealth, exposure to mass media and living number of children

***p < .001

#### Regional patterns

Results of the analyses assessing for regional variations in community level influences on young women’s contraceptive use are presented in Table 4. The findings highlight regional differences and commonalities in community level influences. In African [AOR: 1.12] and European [AOR: 1.31] regions, young women living in communities with higher mean age at first marriage were more likely to report using a modern contraceptive method, but the reverse was true for young women in the Americas [AOR: 0.94] and South East Asia [AOR: 0.71]. The mean age at first birth was positively associated with modern contraceptive use among young women in the African region [AOR: 1.04] but negatively associated with modern contraceptive use in the Eastern Mediterranean region [AOR: 0.78] and South East Asia [AOR: 0.90]. Residing in a community with a higher mean age at sexual debut was associated with lower odds for modern contraceptive use in the African region [AOR: 0.92], European region [AOR: 0.56] and the Americas [AOR: 0.86] but associated with higher odds for modern contraceptive use in South East Asia [AOR: 1.12]. Mass media exposure was negatively associated with modern contraceptive use in the African region [AOR: 0.67], the Americas [AOR: 0.94], South East Asia [AOR: 0.89] but positively associated with modern contraceptive use in Europe [AOR: 1.12] and Western Pacific region [AOR: 1.07].

**Table 4:** Results of multi-level analyses examining regional differences in the effect of community level factors on modern contraceptive use among young women in low- and middle income countries

Community level variable	African Region	Eastern Mediterranean Region	European Region	Americas	South East Asia	Western Pacific Region
	AOR 95% CI	AOR 95% CI	AOR 95% CI	AOR 95% CI	AOR 95% CI	AOR 95% CI
Community mean age at marriage	1.025***	1.15	1.306***	0.942***	0.713***	1.016
	[1.02,1.03]	[0.95,1.39]	[1.12,1.52]	[0.92,0.96]	[0.61,0.83]	[0.87,1.19]
Community mean age at first birth	0.938***	0.782*	0.964	1.018	0.900**	0.909
	[0.93,0.95]	[0.64,0.95]	[0.84,1.10]	[0.99,1.04]	[0.83,0.97]	[0.81,1.02]
Community mean age at sexual debut	0.947***	1	0.558***	0.861***	1.206*	0.995
	[0.94,0.96]	[1.00,1.00]	[0.49,0.64]	[0.84,0.89]	[1.04,1.40]	[0.85,1.16]
Community mean ideal number of children	0.706***	0.588***	0.719***	0.882***	0.342***	0.622***
	[0.70,0.71]	[0.48,0.72]	[0.62,0.84]	[0.84,0.93]	[0.31,0.37]	[0.58,0.67]
Community mean mass media exposure score	1.012**	0.997	1.121*	0.941***	0.897***	1.073*
	[1.00,1.02]	[0.86,1.16]	[1.03,1.23]	[0.92,0.96]	[0.86,0.93]	[1.01,1.14]
Community mean household decision-making autonomy score	1.106***	1.451***	1.042	1.088***	1.169***	1.132**
	[1.10,1.12]	[1.18,1.78]	[0.97,1.12]	[1.07,1.11]	[1.11,1.23]	[1.05,1.22]
Community mean attitudes towards intimate partner violence score	0.985***	0.833**	0.945**	0.857***	1.030*	0.960**
	[0.98,0.99]	[0.75,0.93]	[0.91,0.98]	[0.83,0.89]	[1.00,1.06]	[0.93,0.99]
Community mean years of completed education	1.062***	1.075*	1.171***	1.049***	1.158***	0.877***
	[1.05,1.07]	[1.01,1.15]	[1.07,1.28]	[1.03,1.07]	[1.12,1.20]	[0.82,0.93]
Community mean household wealth score	1	1	1	1.000***	1.000*	1.000**
	[1.00,1.00]	[1.00,1.00]	[1.00,1.00]	[1.00,1.00]	[1.00,1.00]	[1.00,1.00]

All analyses adjusted for age, education level, current marital status, type of place of residence, household wealth, exposure to mass media and living number of children

***p < .001; **p < .05; *p < 1.0

With the exception of South East Asia, young women residing in communities that were more accepting of intimate partner violence were less likely to report using a modern contraceptive method. However, in South East Asia, residing in a community that was more accepting of intimate partner violence was associated with a 3 % increase in the odds for modern contraceptive use. Lastly, a higher education attainment of women in the community was associated with greater odds of modern contraceptive use among young women in Africa [AOR: 1.06], Eastern Mediterranean region [AOR: 1.08], European region [AOR: 1.17], the Americas [AOR: 1.05] and South East Asia [AOR: 1.16] but associated with lower odds for modern contraceptive use among young women in the Western Pacific region [AOR: 0.88].

## Discussion

Despite ongoing efforts to expand access to family planning services, unmet for family planning among young women remains high [4]. Understanding of the contextually relevant community level influences on young women’s contraceptive behavior is necessary for advancing current efforts aimed at reducing unmet need for family planning among young women. Previous research on contraceptive use among young women has mainly focused on individual and institutional level factors and ignored community level influences. A few studies that looked at community factors in contraceptive use s largely focus on women in Africa and combine younger and older women. This paper sought to fill this gap in the literature by identifying the relevant community level factors that shape contraceptive use among young women in Africa and other low and middle-income countries.

With a few notable exceptions, our findings on the global association between the community factors and contraceptive use were largely consistent with the current literature [8, 10, 19, 20]. We found that the education attainment of women in the community was associated with lower odds of modern contraceptive use among young women. While this finding controverts previous reports on the influence of women’s education attainment on reproductive health behavior [8, 10, 19, 20], it is consistent with Ngome and Odimegwu’s findings on the relationship between the community education attainment and contraceptive use among adolescents [7]. Due to the quantitative nature of these data, we are not a position to provide an empirical rationale of this observed relationship. However, Ngome and Odimegwu contend that education does not seem to impact the pro-natalist norms because young women are generally expected to prove their fertility potential through childbearing once they get into a union: contraceptives become a necessity after bearing a child [7]. Nonetheless, there is a need for more research to provide a nuanced understanding of the effects of community education on young women’s reproductive behavior.

Regional patterns in the associations between the community level factors related to mean age at first marriage, mean age at first birth, mean age at sexual debut and exposure to mass media, education attainment of women, attitudes towards intimate partner violence and contraceptive use also emerged. These findings emphasize the contextual nature of the interrelationship between community demographics, socio-cultural norms, and gender dynamics with contraceptive use. These findings also highlight the need to considering both space and time in investigations of community level influences on contraceptive behavior because communities and young women occupy spaces that are continually evolving with political, cultural and social discourses that are unique to each space and time [28, 29]. Young people, in particular, occupy spaces that are rapidly evolving, and the influences of the diverse socio-cultural, gender, economic, political, religious factors on young people’s behavior are unstable, non-linear and reversible [29, 30]. It is thus unrealistic to expect universal trends in the relationship between community level factors and contraceptive as this would imply a universalism in young women’s contraceptive experiences.

Of the several community level factors examined in this paper, the mean ideal family size emerged as the most salient community level predictor of modern contraceptive use among young women. In all regions, young women living in communities with a greater ideal number of children were less likely to report using a modern contraceptive method. This finding could be attributed to the pro-natal attitudes that are prevalent across low and middle-income countries [31, 32]. These pro-natal attitudes – which act as social scripts that women are expected to follow - may discourage use of contraceptive use through increased pressure for young women to prove their fertility [16, 33], or through prevailing fears and misconceptions regarding that modern contraceptive methods may reduce a woman’s fertility [15, 18, 34, 35]. This finding highlights a potential leverage point for structural level interventions aimed at promoting contraceptive use among young women.

### Study limitations

The study has a number of limitations. First, the cross-sectional nature of the DHS data precludes analyses of causal relationships between the community level factors and contraceptive use. Second, the data are heavily weighted towards countries in Africa; as such, they may not provide an accurate representation of other regions that have fewer countries represented within the DHS. Third, as discussed, the PSUs may not be representative of natural community formations and represent artificial sampling blocks. The analysis did not incorporate infrastructural factors (distance to health facility, quality of family planning services), peer-related factors, and ethnic norms/practices that could influence young women’s contraceptive behavior as this is not collected in the DHS. Fourth, our analyses are based on self-report data, which is vulnerable to recall bias and social desirability bias. Some authors have also raised concerns about the quality of data in earlier DHS surveys, particularly with regard to indicators of sexual activity among youths SSA [36]; this could lead to bias in survey estimates. Despite these limitations, these analyses provide valuable insights on the potential mechanism through which community level factors may shape contraceptive use among young women in low and middle-income countries.

## Conclusions

These findings have numerous implications for programs and research aimed at increasing uptake of modern contraceptive methods among women in sub-Saharan Africa. First, they highlight the potential mechanism through which community level factors may shape young women’s contraceptive use. In particular, these highlight the need for community-wide multifaceted programs to address the persistent influence of socio-cultural and gendered norms on uptake of modern contraceptives. Such programs should be implemented in tandem with greater efforts to expand young women’s access to education and employment opportunities, youth friendly sexual reproductive health services, comprehensive health information and life skills training, to promote positive decision-making.

Second, they underscore the need for tailoring and audience segmentation in family planning programs. As indicated, they findings suggest that community level factors may shape contraceptive use in younger and older women differently. Audience segmentation is an important health tool that allows programs to identify relatively homogenous sub-populations with similar characteristics and needs that can be targeted through tailored messages and programs that meet these needs [37, 38].

Lastly, these findings underscore the need for longitudinal studies to enhance our understanding how the dynamic interactions between space and time may shape contraceptive use during the transition to adulthood. Currently, our understanding of contraceptive use among young women is informed by data from cross-sectional studies: this limits our ability to unpack the complexities in women’s experiences, particularly in the context of competing and, at times, conflicting interrelationships between the individual and community level factors that shape women’s FP decision-making processes and experiences. As such, our findings highlight the need for further studies, particularly qualitative and longitudinal studies, to expand our understanding of the complex inter-play between the various individual and community factors that shape women’s contraceptive behavior and how these vary over the life course.

## Abbreviations

AOR: Adjusted odds ratios
DHS: Demographic and Health Surveys
LMICS: Low and middle income countries
mCPR: Modern contraceptive prevalence rate
PSU: Primary sampling unit
WHO: World Health Organization
